# Supplementary material for: Does Older Age Modify Associations between Endocrine Disrupting Chemicals and Fecundability?
Source: Int J Environ Res Public Health. 2022 Jun 30;19(13):8074. doi: 10.3390/ijerph19138074 (PMC9265974; doi:10.3390/ijerph19138074)
Supplement: Supplementary file 1 [file ijerph-19-08074-s001.zip › ijerph-1733327-supplementary.pdf]

Does Older Age Modify Associations between Endocrine Disrupting Chemicals and Fecundability?

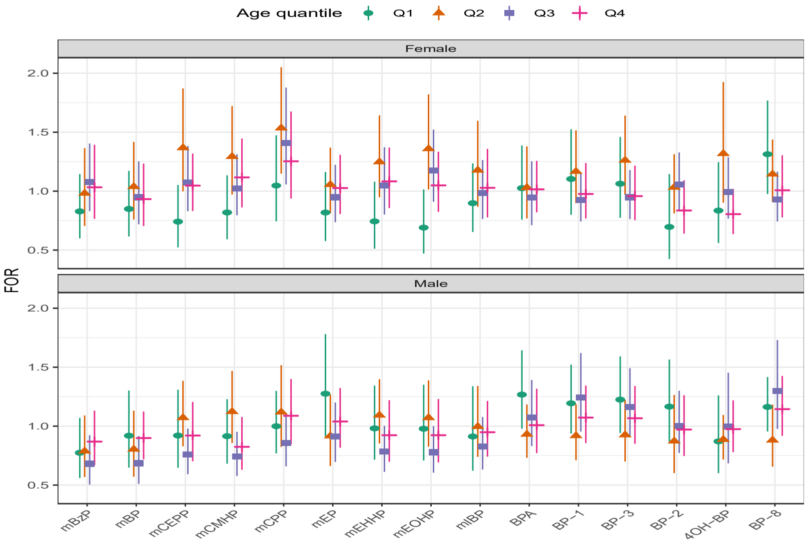

Figure S1. Fecundability odds ratios for phthalate and benzophenone filter metabolites with quantiles of age, separately by sex [females: Q1 (19-26.9 years), Q2 (27-28.9), Q3 (29-32.9), Q4 (33-40), males: Q1 (19-27.9), Q2 (28-30.9), Q3 (31-34.9), Q4 (35-51)]. Models are adjusted for age quantiles, creatinine, cotinine, and BMI.

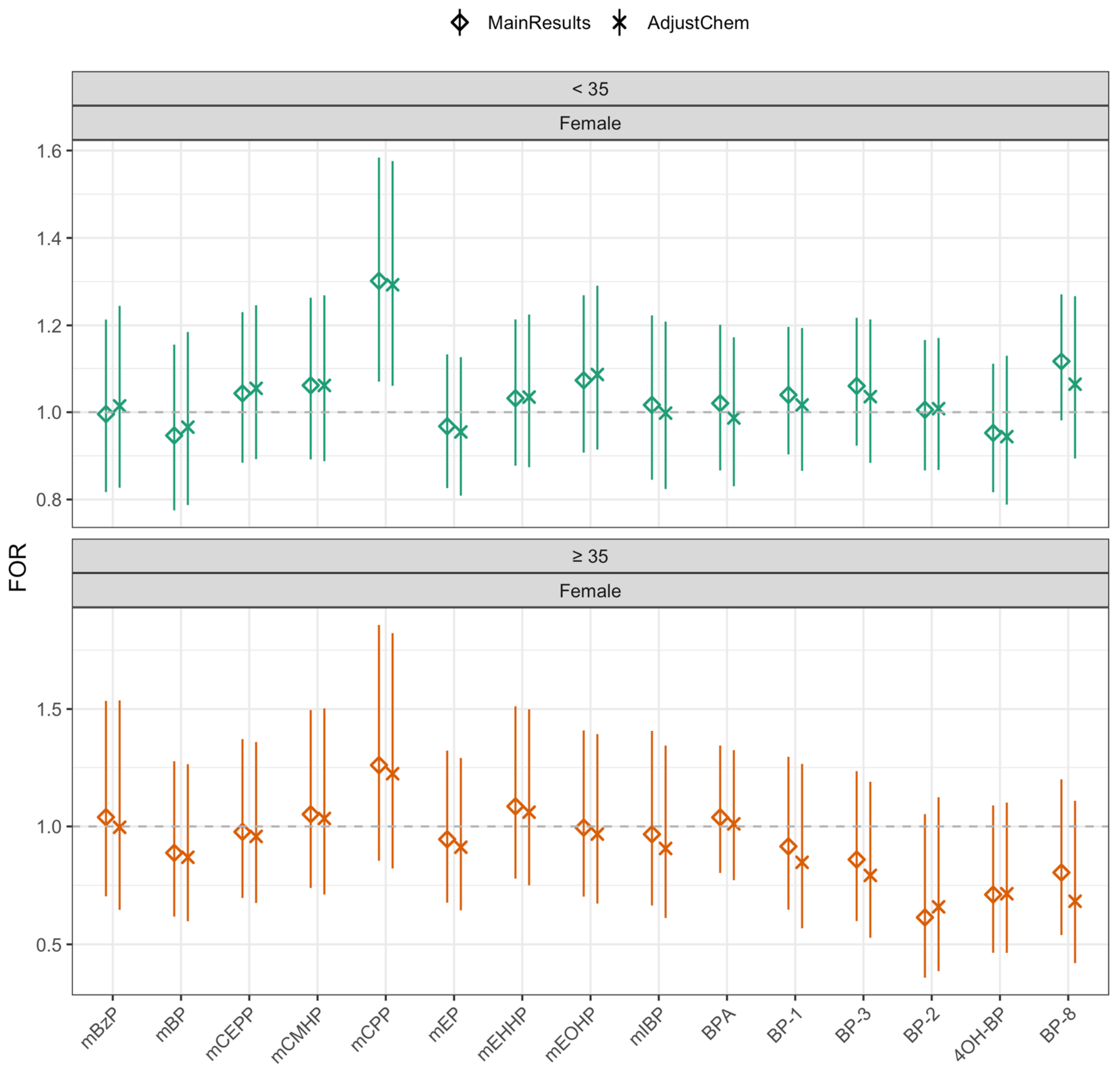

MainResults
AdjustChem
< 35
Female
≥ 35
Female
FOR
1.6
1.4
1.2
1.0
0.8
1.5
1.0
0.5
mBzP
mBP
mCEPP
mCMHP
mCPP
mEP
mEHHP
mEOHP
mIBP
BPA
BP-1
BP-3
BP-2
4OH-BP
BP-8

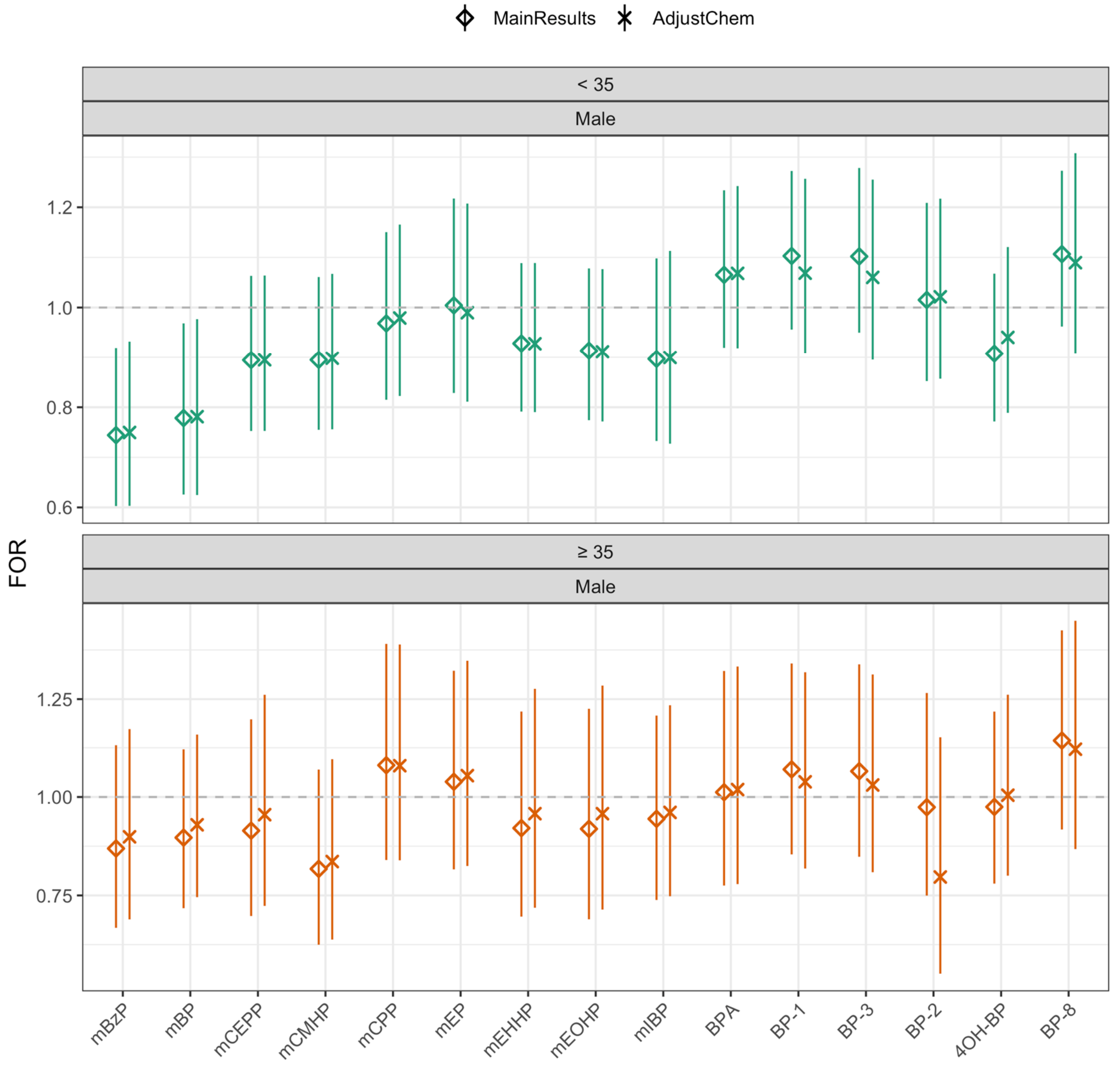

Figure S2. Fecundability odds ratios for both female and male partners at age <35 and age ≥35, controlling for other partner's chemical (AdjustChem) compared to the main results (MainResults). Models are also adjusted for categorical age, creatinine, cotinine, and BMI.
